# Urachal mucinous cystic tumor of low malignant potential in a polymorbid female: a case report and review of the literature

**DOI:** 10.1007/s13691-021-00530-x

**Published:** 2022-01-15

**Authors:** Benjamin Schmeusser, Joseph Wiedemer, Dana Obery, Kaila Buckley, Michael Yu

**Affiliations:** 1 Wright State University Boonshoft School of Medicine, 3640 Colonel Glenn Highway, Dayton, OH 45324 USA; 2 Department of Pathology, CORPath/Riverside Methodist Hospital Associates, Columbus, OH USA; 3 Grant Medical Center, Ohio Health Urology, Columbus, OH USA

**Keywords:** Case report, Urachal cystadenoma, Uro-oncology, Urachus, Mucinous cystadenoma

## Abstract

Neoplasms of the urachus are exceedingly rare, representing 0.17% of all bladder cancers. The mucinous cystic tumor of low malignant potential (MCTLMP) subtype is particularly rare with just 25 previous cases reported in the literature. Although rare, MCTLMPs are important to identify due to potential devastating complications and good cure rates with surgical removal. We present a 43 year old female with a nuanced constellation of comorbidities and confirmed MCTLMP following a workup for abdominal pain and irritative lower urinary tract symptoms. Notably, this tumor did not change in size over a 3-year course of serial imaging prior to surgical excision. This urachal MCTLMP represents roughly the 26th and one of the smallest of its subtype reported in the literature. This case illustrates the diagnosis and management of this rare urachal MCTLMP. Individual patient medical history, clinical considerations, and neoplasm characteristics are examined. Although rare, the potential for increased malignancy and potential complications necessitates surgical management and further investigation by the academic community.

## Introduction

The urachus is an embryologic remnant of the allantois, which connects the anterior dome of the bladder to the umbilicus and rarely persists after early infancy. In the event of incomplete atresia, the urachus has the potential to transform into numerous pathologies including fistula, diverticulum, or tumor. Among the potential pathologies of urachal remnants, urachal neoplasms are exceedingly rare and are of significant interest due to their potentially aggressive invasive potential. Invasive mucinous cystadenocarcinomas have a poor prognosis with only 45% 5 year survival rates. [1, 2]

Isolated cases of urachal neoplasms have presented as pseudomyxoma peritonei and mucosal urinary secretion. Symptomatic presentation varies significantly, but most manifestations include some form of nonspecific abdominal pain [3]. Surgery comprised of excision with partial cystectomy remains the mainstay of care as it is typically curative for noninvasive localized disease [4]. Radiochemotherapeutic intervention has not been studied in randomized multicenter trials and is generally not utilized.

The inner wall of the urachus is lined by epithelium, which may transform into non-glandular, glandular, or mixed neoplasms [4]. Glandular neoplasms are further divided into adenomas, mucinous cystic tumor of low malignant potential (MCTLMP), and adenocarcinoma subtypes [4]. Mucinous cystic tumors, also known as cystadenomas, may be more commonly found in the appendix, ovaries, and pancreas. It is believed that mucinous cystadenocarcinomas can develop from the cystadenomas and MCTLMP [1, 3]. Less than 70 urachal neoplasms have been reported in the literature, among which include just 25 prior known cases of MCTLMP first reported in 2006 [1, 4]. Seven of these reported tumors demonstrated calcifications on imaging [1, 5]. We present the 26th known reporting of MCTLMP.

## Case presentation

A 43 year-old female with a history of newborn gastroschisis, appendectomy, cholecystectomy, colectomy, Roux-en-Y Gastric Bypass, and multiple psychiatric diagnoses had a CT abdomen/pelvis with contrast that incidentally reported a small diverticulum near the bladder dome. A subsequent CT of the abdomen and pelvis without IV contrast 2 years later once again identified the lesion unchanged in size, although suggested a small bladder saccule, diverticulum, or urachal remnant as possible etiologies. Repeat imaging reports failed to identify this abnormality or found it to be stable. Shortly after, the patient was referred to Urology by her primary care physician due to persistent idiopathic lower abdominal pain following an extensive workup. Symptoms at time of urologic consultation included diffuse low- to-mid abdominal pain with associated nausea, vomiting, anorexia, dysuria, and irritative lower urinary tract symptoms.

Cystoscopic evaluation demonstrated a saccular cystic lesion consistent with a urachal remnant cyst with associated tenderness upon compression. 3 years after initial detection on imaging, an open partial cystectomy for excision of the suspicious region was offered as robotic operation was excluded due to prior abdominal surgical history. Intraoperatively, the urachal remnant was palpable and grossly visible on the anterior bladder wall. Abdominal adhesions were extensive as expected from her significant past abdominal surgical history. A partial cystectomy at the dome of the bladder was performed with careful removal of the involved tissue and without any major intraoperative complications.

**Fig. 1:**
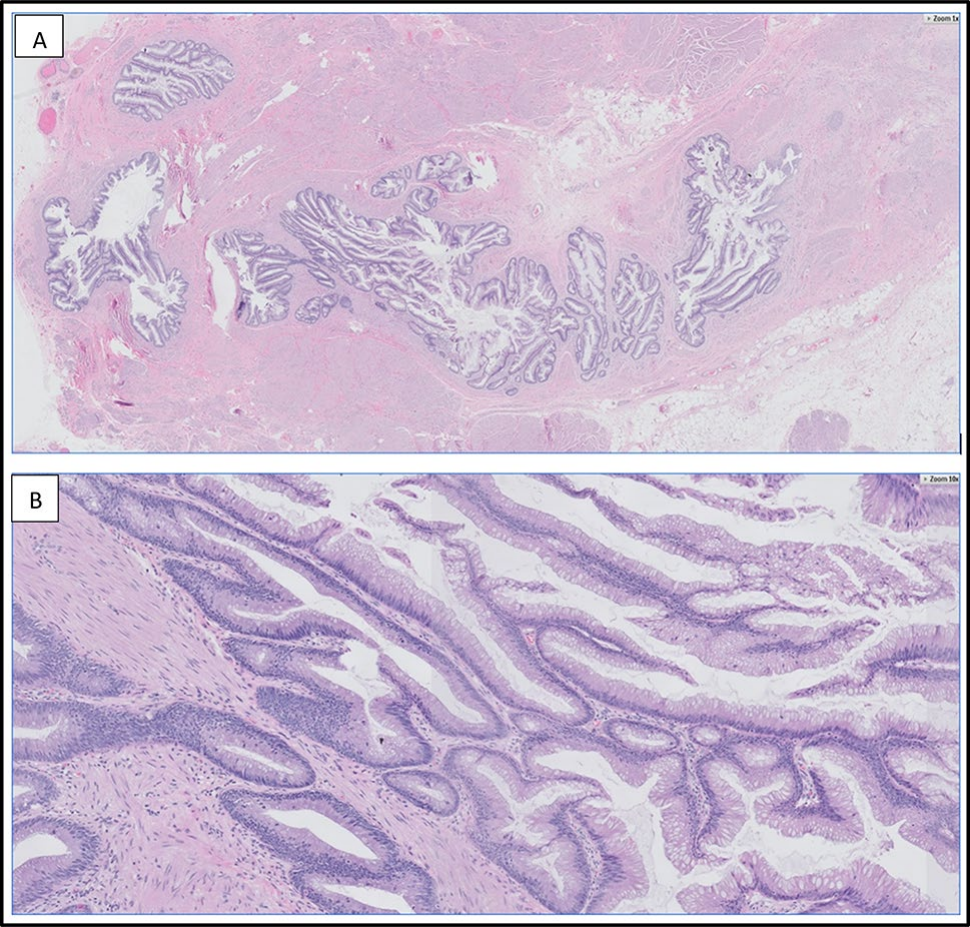
**A**, **B** Histopathologic findings. Multiloculated cystic proliferation composed of a variably complex villous architecture, predominantly lined by a single layer of mucinous epithelium with mild to moderate cytologic atypia. A few mitotic figures are identified. Original magnifications, × 1 (**A**), × 10 (**B**)

**Fig. 2:**
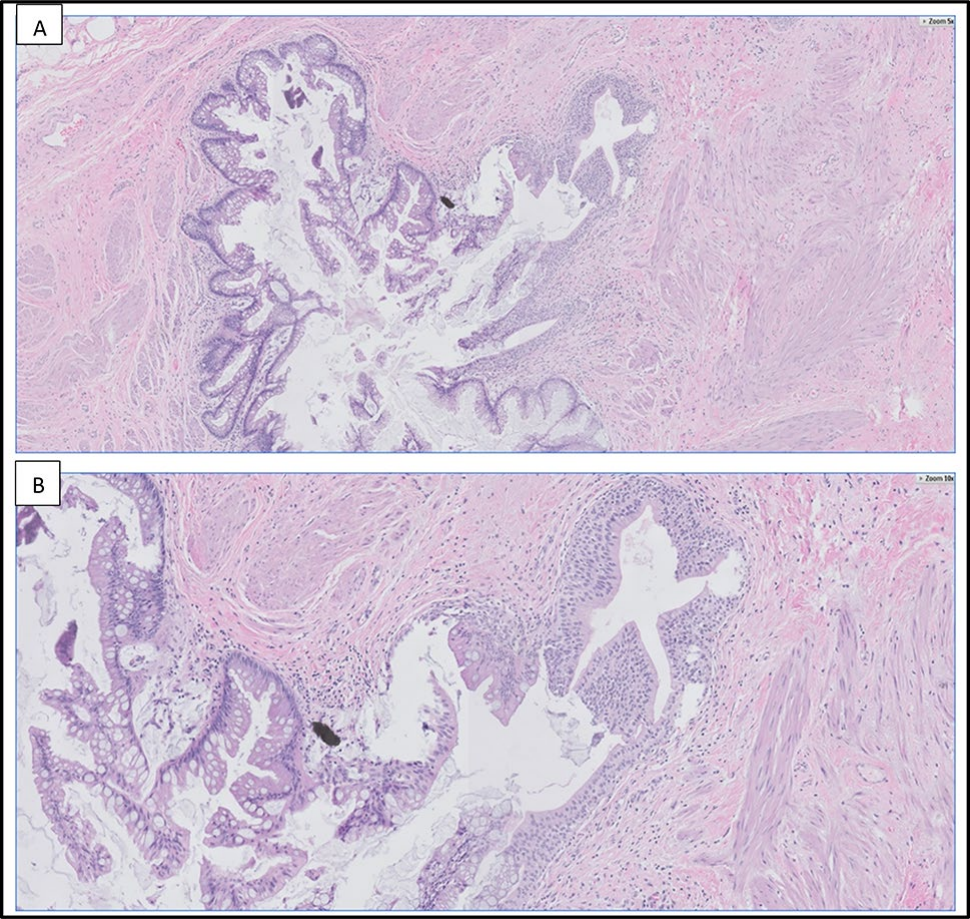
**A**, **B** Histopathologic findings. Mucinous cystic tumor of low malignant potential (MCTLMP). A focus of benign stratified urothelium is visualized on the right, transitioning to atypical mucinous epithelium on the left. Original magnifications, × 5 (**A**), × 10 (**B**)

Gross examination of the excised specimen revealed a 3.5 cm area of nodularity believed to represent the actual size of the tumor. Histopathological analysis revealed cystic structures lined by mucinous epithelium with focal areas exhibiting a pseudostratified cyst lining and at least mild-to-moderate nuclear atypia, consistent with a mucinous cystadenoma of low malignant potential of the urachus (Figs. 1, 2). Histopathological diagnosis was confirmed with a secondary read by Mayo Clinic Laboratories.

Unfortunately, as of roughly two months status-post surgical excision, the patient has reported no symptomatic relief in the symptoms she believed were caused by the excised urachal remnant.

## Discussion

Interestingly, this patient’s history of newborn gastroschisis necessitates consideration of a connection with her subsequent MCTLP. It is unclear if any of the cases in the literature had a known history of gastroschisis. Although the specific pathogenesis of gastroschisis is unclear, hypotheses such as mesenchymal failures, amniotic rupture, persistence of umbilical veins, and vitelline artery disruption should be considered in the conversation of urachal transformation and carcinogenesis. With relation to the urachus, some hypotheses suggest a connection to ventral folding, ventral structures, and the yolk sac [5]. Among the 25 previous reported cases of MCTLMP [4–8], including this patient, the average tumor size is 4.7 cm. Gender does not appear to be a risk factor for MCTLMP occurrence with 13 of the reported cases observed in females and 12 in males.

**Fig. 3:**
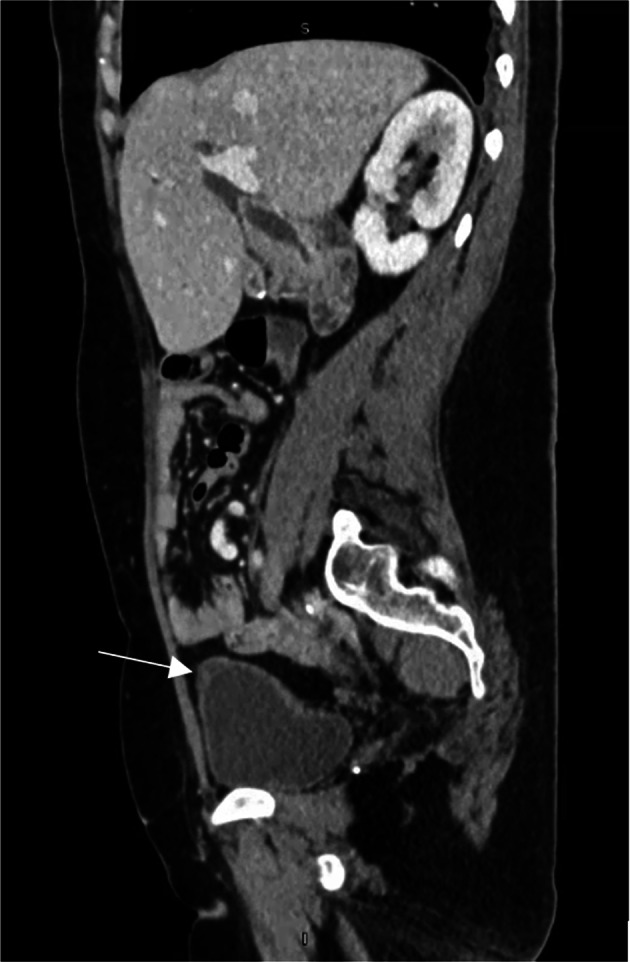
Sagittal view of non-calcified urachal cystic tumor as seen on CT imaging roughly 2 weeks pre-operative noted with white arrow. Tumor remained unchanged in size on imaging from 3 years prior to excision

Furthermore, this patient’s neoplasm was sized at 1.9 × 2 cm on imaging and 3.5 cm on pathological analysis, differing greatly from reported average size of published mucinous urachal cysts of low malignant potential is 5.4 cm (2.0–14 cm) (see Fig. 3) [1]. Given the small size and subtlety of her neoplasm, diagnosis was potentially delayed due to it being missed on imaging and not being palpable on physical exam until her pre-operative cystoscopy. However, given the patient’s extensive medical history and postoperative outcome, it is likely that the urachal neoplasm contributed very little to her constellation of symptoms, if at all. This would be consistent with previous cases reported in the literature, in which clinically meaningful symptomatic presentation is relatively rare, especially in the early stages of this disease [9]. Among the previously reported 25 cases of MCTLMP, 11 were incidentally detected while the remaining cases presenting with nonspecific symptoms with the exception of one case that presented with pseudomyxoma peritonei (Table 1). There are no known cases of MCTLMP that have presented with peritoneal carcinomatosis in the medical literature to date. This patient’s extensive surgical and medical history must be considered as well as multiple complications and diagnoses such as adhesions, bowel obstructions, gynecological issues, among others, were all likely and more common and probable causes of her symptoms. These issues may have delayed her referral, diagnosis, and surgical treatment and it is fortunate that all potential causes were explored.

**Table 1:** Summary of previous cases of urachal MCTLMPs

Reference	Age	Sex	Size (cm)	Diagnosis	Treatment	Symptoms
Shinohara et al. [6]	54	M	6	MCTLMP	Mass excision/partial cystectomy	Pseudomyxoma peritonei
Amin et al. [7]	48	F	8	MCTLMP with intraepithelial carcinoma	Mass/excision/partial cystectomy and umbilectomy	Hematuria, mass
Amin et al. [7]	26	F	2	MCTLMP	Mass excision/partial cystectomy	Suprapubic mass
Amin et al. [7]	74	M	6.5	MCTLMP	Excision of tumor and sigmoid colectomy	Incidental finding
Amin et al. [7]	72	M	0.8	MCTLMP	Mass excision/partial cystectomy	Mucusuria
Amin et al. [7]	74	M	3	MCTLMP	Mass excision/partial cystectomy	Hematuria
Amin et al. [7]	50	F	2.1	MCTLMP	Mass excision/partial cystectomy	Mass
Amin et al. [7]	45	M	3.5	MCTLMP	Mass excision/partial cystectomy	Right lower quadrant pain/hematuria
Amin et al. [7]	58	F	1	MCTLMP	Mass excision/partial cystectomy	Incidental finding
Amin et al. [7]	43	F	2.5	MCTLMP	Mass excision/partial cystectomy	Incidental finding
Amin et al. [7]	40	F	6	MCTLMP	Mass/excision/partial cystectomy & umbilectomy	Incidental finding
Amin et al. [7]	80	F	2.5	MCTLMP	Mass excision/partial cystectomy	Mucusuria
Amin et al. [7]	37	F	NA	MCTLMP	NA	Incidental finding
Amin et al. [7]	29	F	NA	MCTLMP	NA	Bladder dome nodule
Amin et al. [7]	42	F	8	MCTLMP	Mass excision/partial cystectomy	Pelvic mass
Amin et al. [7]	42	F	6	MCTLMP	NA	Midline cystic mass
Amin et al. [7]	36	F	NA	MCTLMP	NA	Incidental finding
Amin et al. [7]	39	M	6.5	MCTLMP	Mass excision/partial cystectomy and umbilectomy	Obstruction and umbilical discharge
Amin et al. [7]	57	M	2.8	MCTLMP with intraepithelial carcinoma	Mass excision/partial cystectomy	NA
Amin et al. [7]	77	F	5.5	MCTLMP	Mass excision/partial cystectomy	NA
Amin et al. [7]	43	M	7	MCTLMP	Mass excision/partial cystectomy and umbilectomy	Incidental finding
Amin et al. [7]	26	M	8	MCTLMP	Mass excision/partial cystectomy	Urgency, abdominal pain
Chahal et al. [8]	37	M	4	MCTLMP	Partial cystectomy, left hydrocelectomy	Incidental finding
Wang et al. [5]	54	M	4	MCTLMP	Mass excision/partial cystectomy and umbilectomy	Incidental finding
Brennan et al. [4]	67	M	9	MCTLMP	Mass excision/partial cystectomy	Incidental finding
Present case (2021)	43	F	3.5	MCTLMP	Mass excision/partial cystectomy	Incidental finding

26 cases of urachal MCTLMP have been found in the literature. Table is sorted by date of publication, and obtainable data on age, sex, size, treatment, and symptoms are included

*MCTLMP* mucinous cystic tumor of low malignant potential, *NA* Not applicable

An interesting aspect of diagnosing MCTLMP and other urachal neoplasms is that diagnosis is based almost entirely on histologic features [3], as described above and demonstrated in Figs. 2 and 3 for our case. Mucinous cystic tumors are classified similarly to mucinous cystic tumors of the ovary, with MCTLMP being defined as a “cystic tumor with areas of epithelial proliferation, including papillary formation and low-grade atypia [9].” Furthermore, MCTLMP linings are more proliferative than cystadenomas and range from flat to tufted and from pseudopapillary to tubulovillous [9].

Immunohistochemical (IHC) analysis was not completed on initial or confirmatory analyses as it does not appear to add any diagnostic value for urachal neoplasms [3]. A study examining the immunohistochemical expression of urachal carcinomas in search of improved delineation of neoplasms found that urachal carcinomas have an overlapping immunoprofile and therefore offered little clinical benefit [10]. However, in cases presenting as a distantly metastasized cystadenocarcinoma, IHC may be necessary to determine tissue origin. Urachal mucinous cystic tumors stain positively for CK20, CDX2, and CK7 in 100%, 80%, and 30% of the cases, respectively, with negative nuclear β-catenin, estrogen receptor, and progesterone receptor staining [3].

Given the potential for complications, growth, and increased malignancy, surgical management of mucinous urachal neoplasms is necessary. MCTLMP should be surgically excised with partial cystectomy followed by periodic imaging and cystoscopy to monitor for recurrence. Future cases concerning for urachal neoplasm should include gross imaging and cystoscopy imaging to contribute to the medical literature, improve characterization of these masses, and improve our overall understanding of these neoplasms. Although data is limited, it appears that the prognosis of MCTLMP is excellent following adequate surgical removal.

## Conclusion

This case illustrates the diagnosis and management of this rare urachal MCTLMP. Individual patient medical history, clinical considerations, and neoplasm characteristics are examined. Although rare, the potential for increased malignancy and potential complications necessitates surgical management and further investigation by the academic community.
